# Intrinsic calf factors associated with the behavior of healthy pre-weaned group-housed dairy-bred calves

**DOI:** 10.3389/fvets.2023.1204580

**Published:** 2023-08-03

**Authors:** Beth B. Riley, Carol-Anne Duthie, Alexander Corbishley, Colin Mason, Jenna M. Bowen, David J. Bell, Marie J. Haskell

**Affiliations:** ^1^Scotland's Rural College (SRUC), Edinburgh, United Kingdom; ^2^Clinical Sciences, The Royal (Dick) School of Veterinary Studies, University of Edinburgh, Edinburgh, United Kingdom; ^3^Dairy Herd Health and Productivity Service, University of Edinburgh, Edinburgh, United Kingdom; ^4^Roslin Institute, University of Edinburgh, Edinburgh, United Kingdom

**Keywords:** activity, behavior, calf/calves, feeding, group-housed, healthy

## Abstract

Technology-derived behaviors are researched for disease detection in artificially-reared calves. Whilst existing studies demonstrate differences in behaviors between healthy and diseased calves, intrinsic calf factors (e.g., sex and birthweight) that may affect these behaviors have received little systematic study. This study aimed to understand the impact of a range of calf factors on milk feeding and activity variables of dairy-bred calves. Calves were group-housed from ~7 days to 39 days of age. Seven liters of milk replacer was available daily from an automatic milk feeder, which recorded feeding behaviors and live-weight. Calves were health scored daily and a tri-axial accelerometer used to record activity variables. Healthy calves were selected by excluding data collected 3 days either side of a poor health score or a treatment event. Thirty-one calves with 10 days each were analyzed. Mixed models were used to identify which of live-weight, age, sex, season of birth, age of inclusion into the group, dam parity, birthweight, and sire breed type (beef or dairy), had a significant influence on milk feeding and activity variables. Heavier calves visited the milk machine more frequently for shorter visits, drank faster and were more likely to drink their daily milk allowance than lighter calves. Older calves had a shorter mean standing bout length and were less active than younger calves. Calves born in summer had a longer daily lying time, performed more lying and standing bouts/day and had shorter mean standing bouts than those born in autumn or winter. Male calves had a longer mean lying bout length, drank more slowly and were less likely to consume their daily milk allowance than their female counterparts. Calves that were born heavier had fewer lying and standing bouts each day, a longer mean standing bout length and drank less milk per visit. Beef-sired calves had a longer mean lying bout length and drank more slowly than their dairy sired counterparts. Intrinsic calf factors influence different healthy calf behaviors in different ways. These factors must be considered in the design of research studies and the field application of behavior-based disease detection tools in artificially reared calves.

## 1. Introduction

Calves born on most dairy farms are separated from their dams and reared artificially (1). Disease is a major concern in these calves, with studies suggesting that 9–48% of artificially reared calves have bovine respiratory disease and 19–46% have diarrhea in the first 9 weeks of life (2, 3). Recently, targets have been set for reductions in both calf mortality and antibiotic use in the first 6 months of life (4). The key to a reduction in the mortality, welfare, financial and production costs of these diseases is early diagnosis. Thus, early diagnosis of disease is an area of considerable research. The use of behavior- based disease detection tools for calves has been increasingly researched (5). It is becoming more common to keep artificially-reared calves in group housing, with the provision of milk through automatic milk feeders. This provides us with the opportunity to record individual calf feeding behaviors. Similarly, the use of animal-mounted sensors is increasing globally. These technologies can aid with disease detection, for example tri-axial accelerometers can be utilized to record activity variables such as lying and standing time (6). Daily lying time and mean lying bout length have been shown to increase in calves with bovine respiratory disease (7), and a reduction in milk consumption has also been seen (8). Calves with neonatal calf diarrhea have been shown to increase lying bout length and decrease lying bout frequency in the days prior to disease development (9) in addition to drinking less milk, and drinking more slowly than their healthy counterparts (10). Recent work has utilized calf behavior to predict both neonatal calf diarrhea (11) and bovine respiratory disease and these models may include calf factors such as live-weight (12).

While there is a considerable body of literature analyzing the effects of group housing, nutrition, and disease on calf behavior in the pre-weaned period, there is very little in the published literature regarding the effects of calf factors such as age, live-weight, and breed on behavior in the pre-weaned period. Many previous studies have been designed to control factors such as age (13), sex (7, 8) and breed (6), frequently by only using one breed or sex. Thus, there is very little information to support our assumptions that these intrinsic calf factors require controlling for or as to which factors should be used in predictive models. For instance, age of the calf has been shown to influence behavior in the limited amount of literature available. Calves had more lying bouts and less time at the feeder at 40 days of age than at 26 days of age (14). Standing time has been shown to increase by 0.52 min per day of age (15). Age has also been shown to influence behavior during transport of calves < 10 day old (16).

While there is literature available on the effect of breed and sex on, for example, flight speed, behavior in the handling chute (17), avoidance behaviors (18) and curiosity (19), there is no research into the effects of calf breed on lying and feeding behaviors in the pre-weaned period. One previous study has analyzed differences in activity variables between sexes with female calves spending more time walking than their male counterparts but no differences in lying or standing time (20). Another factor that affects calf behavior is the social environment. Calves that were group-housed from 3 days of age had more social interactions and performed more play behaviors than those grouped from 7 or 14 days of age (21). However, there was no information given on activity and feeding behaviors.

A variety of environmental factors may also influence calves, including heat stress, with calves in a non-shaded environment changing posture more frequently than those in the shade (22). However, to the authors' knowledge, there is no published information on whether the behavior of pre-weaned calves is affected by season in the UK climate. One study did show that calves were more likely to seek refuge at higher induced wind speeds (23), but there was no work looking at transitions between lying and standing or time spent lying or standing.

Maternal factors may also influence the calf. The pre-natal environment may influence factors such as birthweight, dystocia, immune function, and calf survival (24). Calves born to dams suffering from metabolic stress in late lactation have lower birthweights and an increased basal inflammatory response compared to calves born to dams experiencing a lower level of metabolic pressure (25). Another study found increased inflammation and metabolic stress in calves born to cows with a low body condition score 24 days prior to calving (26). Heifers born to primiparous mothers were found to be at higher risk of both mortality and culling prior to first calving than those born to multiparous mothers (27). Calf lying behavior may be influenced by maternal management during the dry period with calves born to cows kept at pasture during the dry period having longer lying periods than calves born to cows kept indoors but exercised daily (28). Calves born to dams that suffered heat stress in late lactation have altered standing variables when compared to calves born to cows that did not suffer from heat stress in late lactation (29). Furthermore, these effects may be long lasting, as heat stress in late gestation has been found to effect the milk production of both the heifer born from that gestation and the production of the heifer's daughter (30). Disease during the lactation where the heifer was conceived is associated with an increase in age at first calving and a reduction in second lactation fat yield (31).

As detailed above, the evidence suggests that several calf, dam, and environmental characteristics can affect behavior. As changes in behavior are being used in studies that aim to detect disease, we need to determine what factors are associated with key behaviors and what the size of these associations are, so that they may be accounted for in future study designs. To identify calf factors that may need to be controlled for in comparison studies or included in predictive models, an understanding of how these factors are associated with feeding and activity variables in a healthy pre-weaned calf is needed. This study aims to address this gap by analyzing the association of intrinsic calf factors with behavior in healthy pre-weaned artificially reared calves.

## 2. Materials and methods

### 2.1. Animals and housing

This work was conducted as part of the wider WELL-CALF project (UKRI project ref. 105143) under the approval of the Animal Experiment Committee of SRUC (DAI AE 10-2020) and in accordance with the Animals (Scientific Procedures) Act 1986. Calves were sourced from two sub-herds (A and B) that are kept on a single holding at SRUC Dairy Research and Innovation Center, Crichton Royal Farm, Dumfries, UK. Calves born between 07.24.20 and 01.11.21 were included in the trial. Due to a long-term trial examining genetic and environmental interactions, calves from sub-herd A came from two distinct Holstein lines: a commercial line and a high yielding line. Only male Holstein calves were included from this farm. Sub-herd B consisted of a mixture of Holstein lines. Of the 114 calves recruited into the study, forty-six had beef breed sires (Aberdeen Angus, Belgian Blue, or Longhorn) and sixty-eight had Holstein sires.

All calves received four liters of pasteurized, quality-checked thawed colostrum as soon as possible after birth. As is normal practice for this farm, calves were removed from their dam in the first 24 h of life and placed in individual straw bedded hutches. Here calves received 3 liters of reconstituted acidified milk replacer in a bucket with a teat twice daily (*Maximum* + *acidified, Carrs Billington Ltd.*, crude protein 24%, crude oils and fats 20%, crude ash 7.5%, calcium 0.9%, sodium 0.5%, phosphorus 0.7%, mixed to 15%). Starter pellets (compounded for the farm by *Forfarmers UK Ltd. Rougham*, dry matter 82.2%, crude protein 18%, crude oils and fats 4.5%, crude fiber 10%, crude ash 10.5%, Calcium 2%, decoquinate [*Deccox 6%, Zoetis UK Ltd. Leatherhead Surrey, 50 mg/kg*)\ and water were offered in buckets *ad libitum*. On the advice of the farm's veterinary surgeon all calves born between 24.07.20 and 31.12.20 received halofuginone (*Halocur 0.5 mg/ml oral solution for calves, MSD Animal Health, Walton, Milton Keynes*, 100 μg/kg live-weight by mouth) once daily from the first to sixth day of life. For calves born after 01.01.21, this was changed to paromomycin sulfate given in milk between days zero and six of life (*Parofor, Huvepharma NV, Antwerp, Belgium*, 10 g/calf/day).

Calves were moved into groups of twelve to fourteen calves and entered the study at six to thirteen days of age (mean 8.7 days, standard deviation (SD) 2 days, median 9 days). Group pens were straw-bedded and consisted of a roofed pen (5.1 m x 5.1 m) and an igloo (3.9 m x 4.4 m, 2.2 m high) (stocking density 3.1–3.6 m^2^/calf). Calves were offered *ad libitum* calf starter (as above) in a trough and straw in a hay rack. Each group pen of twelve to fourteen calves had access to one teat on a single automatic calf feeding unit (c*ustom built for this calf unit, BioControl Norway As, Grimstad Gård, Norway*). Milk was mixed as above and calves were allowed seven liters (regardless of age) between 00:00 and 23:59. However, if they had not reached this maximum, they could carry over 1.4 liters into the next day. The maximum meal size was 1.4 liters with the day being split into five feeding periods with an additional feed becoming available at the start of each period. Water was available *ad libitum*. Both water and milk lanes included a weigh-scale, and a live-weight was recorded during each visit. A daily mean of these recorded live-weights was calculated to create the live-weight variable, as previously validated (32).

Wisconsin calf health scoring was carried out as described by McGuirk (33). Scoring was done daily by three trained technicians. The technicians were trained together in the scoring by a veterinary surgeon, and then scored a pen of ten calves together. At the end of training consistency between scorers was checked by them independently scoring a separate pen of calves. To verify the consistency between scorers across the entire dataset, scores from all scorers were analyzed on the first day present in the data set for all calves (14 days of age). The largest dataset would have allowed for any bias to be detected. However, a Kruskal-Wallis test showed no significant difference between technicians (Kruskal-Wallis chi-squared = 2.31, df = 2, *p* = 0.315). Fecal scores were dropped from the scoring system as this measure is difficult in group-housed calves and diarrhea was not the central aim of the study. During health scoring, all calves were closed into the front of the pen for the period in which scoring took place. The farm staff treated calves independent of the Wisconsin score. If a calf had a rectal temperature of ≥39.5°C or clinical signs of respiratory disease for 2 days or more, then the farm staff were informed.

The automatic milk feeder recorded the time of each visit where milk was consumed, the length of that visit, the volume of milk consumed at the visit and the speed at which the calf drank the milk. This was used to calculate daily values for total time at milk feeder (minutes), total milk visits (n), mean milk visit length (minutes), mean milk drinking speed (g/s), volume of milk drunken/day (ml) and mean milk per visit (ml) for each calf.

At time of entry to the group pen, a tri-axial accelerometer was placed on the right hind leg (*IceQube, IceRobotics, Edinburgh*). The IceQube accelerometer was previously validated in group housed calves that were 55 days old (34). The Motion Index (MI) is a proprietary indicator calculated by IceRobotics from the raw accelerometer data and has no units. The data output for the IceQube gave the number of transitions up (from lying to standing), the number of transitions down (from standing to lying), the time standing, the time lying and the motion index for each 15-min period. This was used to calculate daily values for the daily lying time (minutes), daily standing time (minutes), daily lying bouts (n), daily standing bouts (n), total daily motion index, mean lying bout length (minutes), mean standing bout length (minutes) and mean motion index per standing bout for each calf.

### 2.2. Data handling

Initial data handling was performed in Microsoft Excel with activity, feeding and health score data all being converted to this format. The day of entry to the pen was removed from all data to ensure full 24-h periods were available for analysis. Days where sick calves had been removed to individual pens for additional nursing were also removed. Only data from the day after entry to the group pen until the day prior to the commencement of weaning (39 days of age) was included for analysis. Calves were moved daily by technicians for health scoring thus 2 h of data were removed from each day's activity data. A period was chosen that accounted for 90% of the scoring times (7:15–9:15 a.m.). Data processing was carried out in R (35) using the R Studio graphical interface (*R studio, Boston, Massachusetts)* using the tidyverse package (36). Descriptive statistics were calculated in R studio using the psych package (37).

From the 114 calves on trial, seven were excluded from analysis. Reasons for exclusion were as follows; reluctant to drink (*n* = 1), mortality due to bloat (*n* = 1), congenital heart defect (*n* = 1), ear tag infection/iatrogenic ear droop (*n* = 3) and a faulty IceQube (*n* = 1). Another nine calves did not remain in the pen until weaning. Reasons for this included mortality (*n* = 3), removed to individual pen for nursing care (*n* = 4), lameness (*n* = 1) and ear swelling (*n* = 1). Eight calves had missing activity data due to equipment problems or calves not spending sufficient time in proximity to the base station (4.8% of total calf days). 3 days of feeding data were missing due to technical problems. One calf had no birthweight recorded.

A daily total Wisconsin score for each calf was calculated using the rectal temperature, cough, and nasal discharge scores and whichever was highest of either the ear or eye scores as previously described (33). Healthy calves were identified as those with a Wisconsin score of ≤ 3. This threshold was chosen based on that used by McGuirk and Peek (38) for healthy ( ≤ 3), intermediate (4) and diseased calves (≥5). For this study the aim was to create a balanced data set including only periods of time where a calf could be deemed as healthy without evidence of developing or recovering from disease. To do this, 3 days either side of any Wisconsin score >3, temperature score >2 or veterinary treatment were removed from the dataset. Calves with at least ten consecutive healthy days were included (*n* = 31) and those with below ten consecutive health days (*n* = 59) removed. Where the healthy period was longer than ten days, the middle ten days of the healthy period were taken. The breakdown of herd, sex, and sire breed type for these thirty-one calves is shown in the (Supplementary Table 1). From these 310 calf days, seven calf days (4 calves) of activity data were missing due to technical problems. One calf had no birthweight recorded but was still included in the analysis.

### 2.3. Data analysis

Mixed modeling on the 310 calf days in the data set was undertaken using the lme4 package of R (39). To determine which factors to include in the multivariable model for each behavior, a univariable linear mixed effects model was constructed for each calf behavior by intrinsic factor combination (Table 1). Animal identity was nested within group as a random effect to allow for repeated measures on each calf. The factors analyzed were sex, live-weight, age, sire breed type, birthweight, season of birth, age when transferred to the group pen and dam lactation. Dam lactation was classified as 1, 2, or 3+ and sire breed type as beef or dairy. Calves born in July and August were classed as being born in summer, those born in September, October and November as born in autumn and those born in December and January as born in winter. Sub-herd was not tested, as of the eighteen calves from sub-herd A included in the study seventeen were male Holstein calves (Supplementary Table 1). *P*-values were calculated using the summary() function in the lmerTest package (40). All variables that had a *p* < 0.2 in the univariable analysis were taken forward into the multivariable analysis. A forward stepwise approach was used, and final model selection was carried out based on the Akaike information criterion corrected for small sample sizes (AICc, aictab(), AICcmodavg) (41). Where highly correlated variables were both significant at the univariable stage (i.e., age and live-weight and live-weight and birthweight see Supplementary Table 1), model building was performed with each of those variables separately, with the final model selected using AICc. Residuals were visually checked using a q-q plot produced using the DHARMa package (42) where appropriate. For those behaviors where residuals were not normally distributed, then a log or square transformation was conducted. Mean lying bout length, mean standing bout length, total milk visits and mean milk visit length were analyzed using a natural log transformation. Mean milk drinking speed was analyzed using a square transformation. No transformation was necessary for daily lying bouts, daily standing bouts, total daily MI, mean MI per standing bout and mean milk per visit. The summary() function was used to calculate estimates and *p*-values for each model, the anova() function to calculate the numerator degrees of freedom (ndf) and denominator degrees of freedom (ddf), the confint() function was used to calculate the confidence intervals (CI) of the estimates (39) and where necessary, the exp() or sqrt() functions were used to back transform estimates and confidence intervals. The emmeans package (43) was used to calculate estimated marginal means (EMM), pairwise comparisons, *p*-values and the associated standard errors. EMM are calculated from the model, considering the other variables within the model, and give each cell in the reference grid equal weights which is advantageous in unbalanced datasets. These were then plotted using ggplot (44) to allow visualization of the results.

**Table 1:** The effects of calf and dam characteristics on activity and feeding behaviors.

**Behavior**	**Sire breed type**	**Season of birth**	**Sex**	**Birthweight**	**Age at inclusion**	**Age^d^**	**Live-weight^d^**	**Dam lactation**
Daily lying bouts^a^	0.137^*^	0.043^*^	0.143^*^	0.048^*^	0.528	0.359	0.840	0.591
Daily standing bouts^a^	0.131^*^	0.030^*^	0.160^*^	0.045^*^	0.525	0.492	0.991	0.625
Mean lying bout length^a^	0.077^*^	0.089^*^	0.063^*^	0.073^*^	0.647	0.562	0.859	0.743
Mean standing bout length^a^	0.263	0.035^*^	0.612	0.065^*^	0.522	0.005^*^	0.016^*^	0.502
Total daily MI^a^	0.694	0.260	0.060^*^	0.326	0.120^*^	0.002^*^	0.054^*^	0.284
Mean MI per standing bout^a^	0.718	0.423	0.571	0.706	0.027^*^	0.005^*^	0.072^*^	0.483
Mean milk drinking speed^a^	0.109^*^	0.180^*^	0.181^*^	0.692	0.395	0.011^*^	0.044^*^	0.275
Total milk visits^a^	0.558	0.364	0.702	0.121^*^	0.851	0.064^*^	< 0.001^*^	0.064^*^
Mean milk visit length^a^	0.151^*^	0.600	0.596	0.151^*^	0.940	0.012^*^	< 0.001^*^	0.857
Mean milk per visit^a^	0.346	0.498	0.278	0.006^*^	0.294	0.270	0.004^*^	0.051^*^
Daily milk allowance consumed^b^	0.350	0.687	0.022^*^	0.852	0.355	0.062^*^	0.031^*^	0.692
Daily lying time^c^	0.366	0.062^*^	0.155^*^	0.355	0.370	0.777	0.319	0.484
Daily standing time^c^	0.411	0.066^*^	0.160^*^	0.363	0.391	0.229	0.310	0.483
Total time at milk feeder^c^	0.414	0.218	0.711	0.852	0.891	0.598	0.779	0.328

*P-*values for univariable models are shown and factors taken forward for multivariable model building are depicted with an ^*^.

a Linear mixed model.

b Binomial generalized linear mixed model.

c Multinomial generalized linear mixed model.

d Age and live-weight for each of the individual 310 calf days.

As these calves were fed a restricted level of milk, there was an extreme right skew to the data for the daily milk volume consumed and hence this data was not suitable for analysis using a linear mixed model. Thus, the data was converted into a binary variable of whether a calf drank its daily milk allowance or not. Calves that had drunk over 6.5 L were classed as eating their full daily allowance and coded as 1 and those eating less than this were coded as 0 for each day. This data was then analyzed using a binomial generalized linear mixed model. Model building and selection was conducted using the same method as the models above. Group and animal number were included as nested random effects. Overdispersion was checked for using the equation suggested by Bolker (45). The odds ratio and CI were then calculated using base R (35).

For behaviors where the model residuals were non-normal after transformation, multinomial mixed modeling was conducted using the generalized linear mixed modeling function in GenStat (*19*^*th*^ *Edition, VSN International, Hemel Hempstead, Hertfordshire, 2020*). Model building was conducted using a forward stepwise approach using the variables that had *p* < 0.2 at the screening stage. Daily lying time, daily standing time and total time at milk feeder were analyzed in this way.

## 3. Results

For the thirty-one calves included in the analysis, the mean age was 27.7 days (range 11–39 days) and the mean live-weight 56.5 kg (range 41.9–79.9 kg). For further descriptive statistics of the intrinsic factors, please see, Supplementary Table 3. Descriptive statistics for each feeding and activity variable are available in Supplementary Table 3.

Univariable analysis identified thirteen out of fourteen behaviors as suitable to move forward to the model building stage (Table 1). The factors tested were sire breed type, season of birth, sex, birthweight, age at inclusion into the group pen, age, live-weight, and dam lactation number. All factors tested were taken forward to the model building stage for at least two of the thirteen behaviors that underwent multivariable analysis. The multinomial analysis of total time at milk feeder yielded no variables with a *p* < 0.2 at the screening stage and therefore this analysis was not taken forward to the multivariable stage. The forward model building for daily standing time yielded only a univariable result at *p* = 0.066 for season of birth (Wald = 6.06, F = 3.03. ndf = 2, ddf = 26) as the multivariable model did not converge, therefore this model was disregarded.

### 3.1. Live-weight had a major significant association with milk feeding behaviors

**Figure 1:**
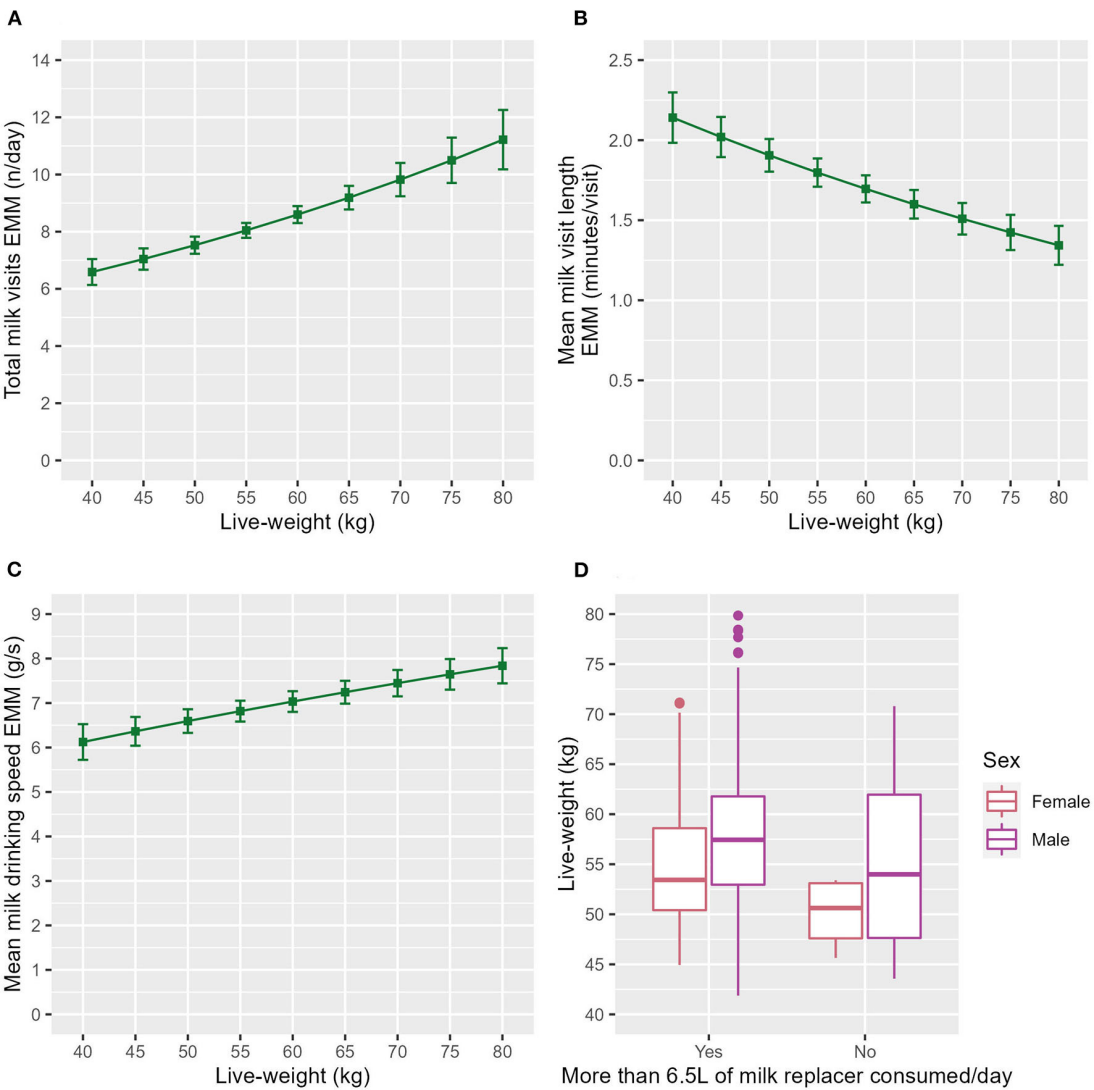
The estimated marginal means (EMM) of the **(A)** total milk visits, **(B)** mean milk visit length and **(C)** mean milk drinking speed in relation to live-weight. Error bars denote the standard error. **(D)** Box and whisker plot showing the raw data live-weight range of both sexes for the calves that had drunk their milk allowance each day and those that had not.

Heavier calves visited the milk machine more frequently (ndf = 1, ddf = 84.3, *t* = 3.819, *p* < 0.001, Figure 1A) and for a shorter visit length (ndf = 1, ddf = 200.3, *t* = −3.673, *p* < 0.001, Figure 1B) than their lighter counterparts. Heavier calves drank faster than their lighter counterparts (ndf = 1, ddf = 129.7, *t* = 2.770, *p* = 0.006, Figure 1C) and were more likely to drink their milk allowance (z = 2.430, *p* = 0.016, **Table 3**). This is illustrated in Figure 1D, which shows that the calves that had consumed their daily milk allowance were heavier on average.

### 3.2. Age was associated with activity variables

**Figure 2:**
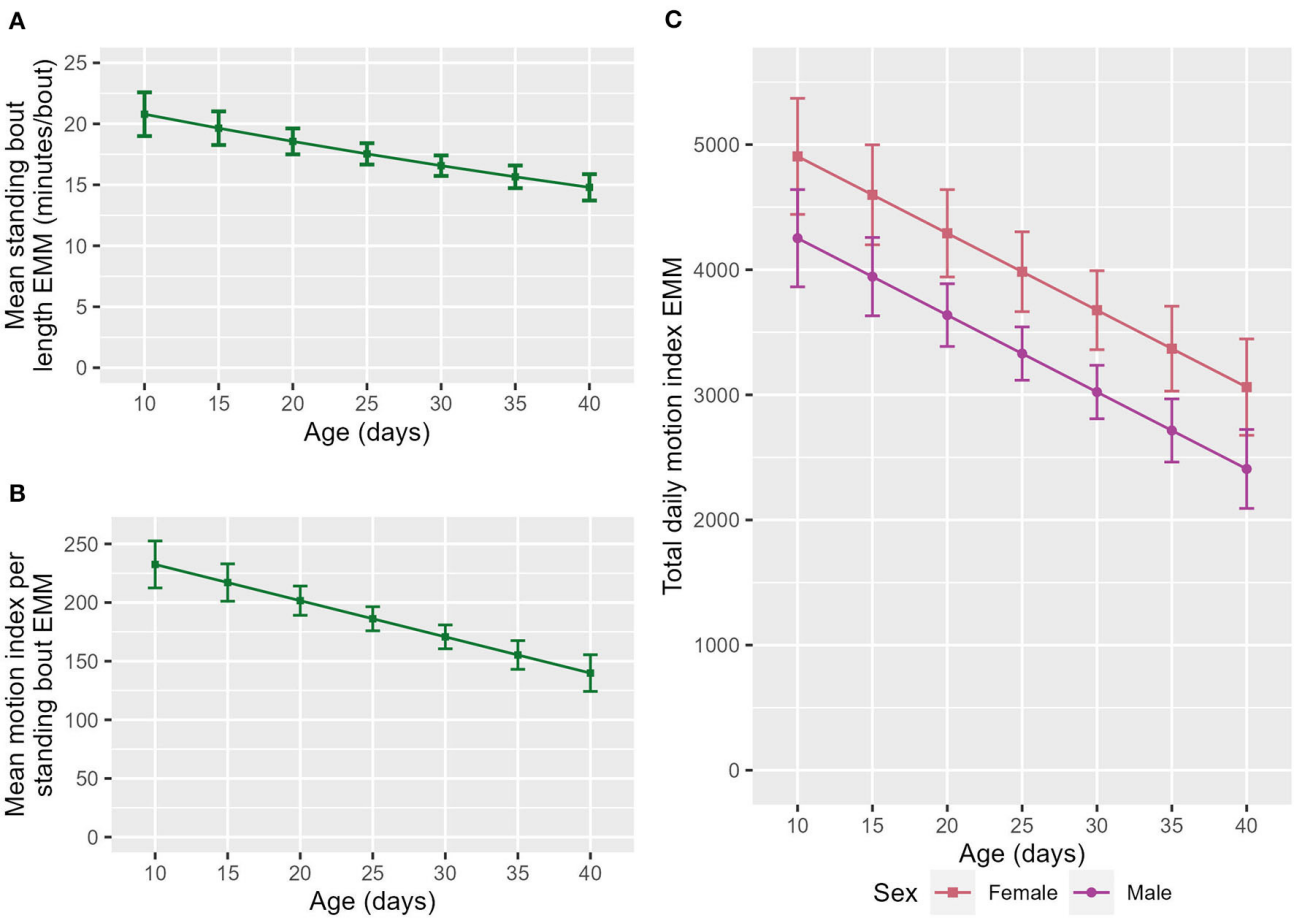
The estimated marginal means (EMM) of the **(A)** mean standing bout length and **(B)** motion index per standing bout in relation to age. **(C)** The EMM of total daily motion index in relation to age and sex. Error bars denote the standard error.

Older calves had a shorter mean standing bout length (ndf = 1, ddf = 224.2, *t* = −2.797, *p* = 0.06, Figure 2A) than their younger counterparts. Calves also had a lower total daily MI (ndf = 1, ddf = 145.5, *t* = −3.396, *p* < 0.001, Figure 2C) and mean MI per standing bout (ndf = 1, ddf = 114.1, t = −3.267, *p* = 0.001, Figure 2B) as they got older.

### 3.3. Season of birth was associated with activity variables

**Figure 3:**
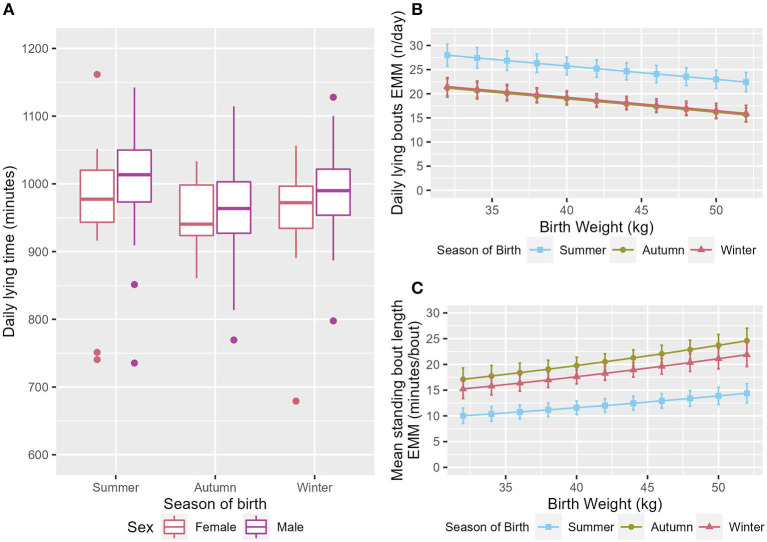
**(A)** Box and whisker plot of the raw data for daily lying time showing the range of values across the three seasons and both sexes. The EMM of the **(B)** daily lying bouts, **(C)** mean standing bout length in relation to season and birthweight. Error bars denote the standard error.

Calves born in summer had a longer daily lying time, with calves born in autumn having the lowest daily lying time (ndf = 2, ddf = 3.98, Wald = 7.94, *p* = 0.031, Figure 3A). Both autumn- and winter-born calves had fewer lying bouts/day than summer-born calves (ndf = 2, ddf = 9.7, *t* = −3.259, *p* = 0.009, and *t* = −3.045, *p* = 0.015 respectively, Figure 3B). Calves born in both autumn and winter also had fewer standing bouts each day than those born in summer (ndf = 2, ddf = 9.7, *t* = −3.504, *p* = 0.006, and *t* = −3.206, *p* = 0.012 respectively, Supplementary Figure 1). Calves born in autumn and winter had a longer mean standing bout length than calves born in summer (ndf = 2, ddf = 8.4, *t* = 4.135, *p* = 0.002, and *t* = 3.263, *p* = 0.012 respectively, Figure 3C). Season was maintained in the model for mean milk drinking speed as it improved fit but was not significant (ndf = 2, ddf = 8.71, F = 2.264, *p* = 0.162, Table 2).

**Table 2:** The effect of calf and dam characteristics on activity and feeding behaviors of healthy pre-weaned artificially reared calves.

**Behavior**	**Fixed effect**	**Level**	**Effect size**	**Confidence interval**	***P*-value**
Daily lying bouts (*n*)	Season of birth	Summer	Reference^b^	Reference	Reference
		**Autumn**	**−6.8**	**−10.6 to** **−3.0**	**0.009**
		**Winter**	**−6.5**	**−10.5 to** **−2.6**	**0.016**
	**Birthweight**		**−0.3**	**−0.5 to** **−0.1**	**0.023**
Daily standing bouts (*n*)	Season of birth	Summer	Reference	Reference	Reference
		**Autumn**	**−7.2**	**−11.0 to** **−3.4**	**0.006**
		**Winter**	**−6.7**	**−10.7 to** **−2.9**	**0.012**
	**Birthweight**		**−0.3**	**−0.5 to** **−0.1**	**0.021**
Mean lying bout length (minutes)	Sire breed type	Beef	Reference	Reference	Reference
		**Dairy**	**−0.8**	**−0.7 to** **−0.9**	**0.008**
	Sex	Female	Reference	Reference	Reference
		**Male**	**1.2**	**1.1–1.4**	**0.009**
Mean standing bout length (minutes)	Season of birth	Summer	Reference	Reference	Reference
		**Autumn**	**1.7**	**1.4–2.1**	**0.002**
		**Winter**	**1.5**	**1.2–1.9**	**0.012**
	**Age**		**−1.0**	**−1.0 to** **−1.0**	**0.006**
	**Birthweight**		**1.0**	**1.0–1.0**	**0.04**
Total daily MI	Sex	Female	Reference	Reference	Reference
		Male	**–**654.0	**–**1,360.5–3.3	0.065
	Age at inclusion		**–**125.9	**–**276.4–34.5	0.121
	**Age**		**−61.5**	**−96.2 to** **−25.8**	**< 0.001**
Mean MI per standing bout	**Age at inclusion**		**−11.2**	**−18.9 to** **−2.2**	**0.007**
	**Age**		**−3.1**	**−4.9 to** **−1.0**	**0.001**
Mean milk drinking speed (g/s)	Season of birth	Summer	Reference	Reference	Reference
		Autumn	2.1	**–**4.2–4.2	0.572
		Winter	3.8	0.7–5.3	0.112
	Sire breed type	Beef	Reference	Reference	Reference
		**Dairy**	**4.0**	**2.7–5.1**	**0.003**
	Sex	Female	Reference	Reference	Reference
		**Male**	**−3.9**	**−4.9 to** **−1.5**	**0.005**
	**Live-weight**		**0.8**	**0.4 to 1.0**	**0.006**
Total milk visits (n)	**Live-weight**		**1.0**	**1.0–1.0**	**< 0.001**
Mean milk visit length (minutes)	**Live-weight**		**−1.0**	**−1.0 to** **−1.0**	**< 0.001**
Mean milk per visit (ml)	**Birthweight**		**−13.9**	**−26.3 to** **−1.8**	**0.041**
	Dam Lactation	1	Reference	Reference	Reference
		2	68.8	−71.7–209.6	0.363
		3+	−35.9	−185.1–110.4	0.648

The results shown are from the final linear mixed models. Factors with *p* < 0.05 are shown in bold.

^a^The results shown for the models where a transformation was used have been back transformed.

b The level of a categorical variable that the model uses as the “baseline.”

### 3.4. Male calves tended to be less active and drank less

**Figure 4:**
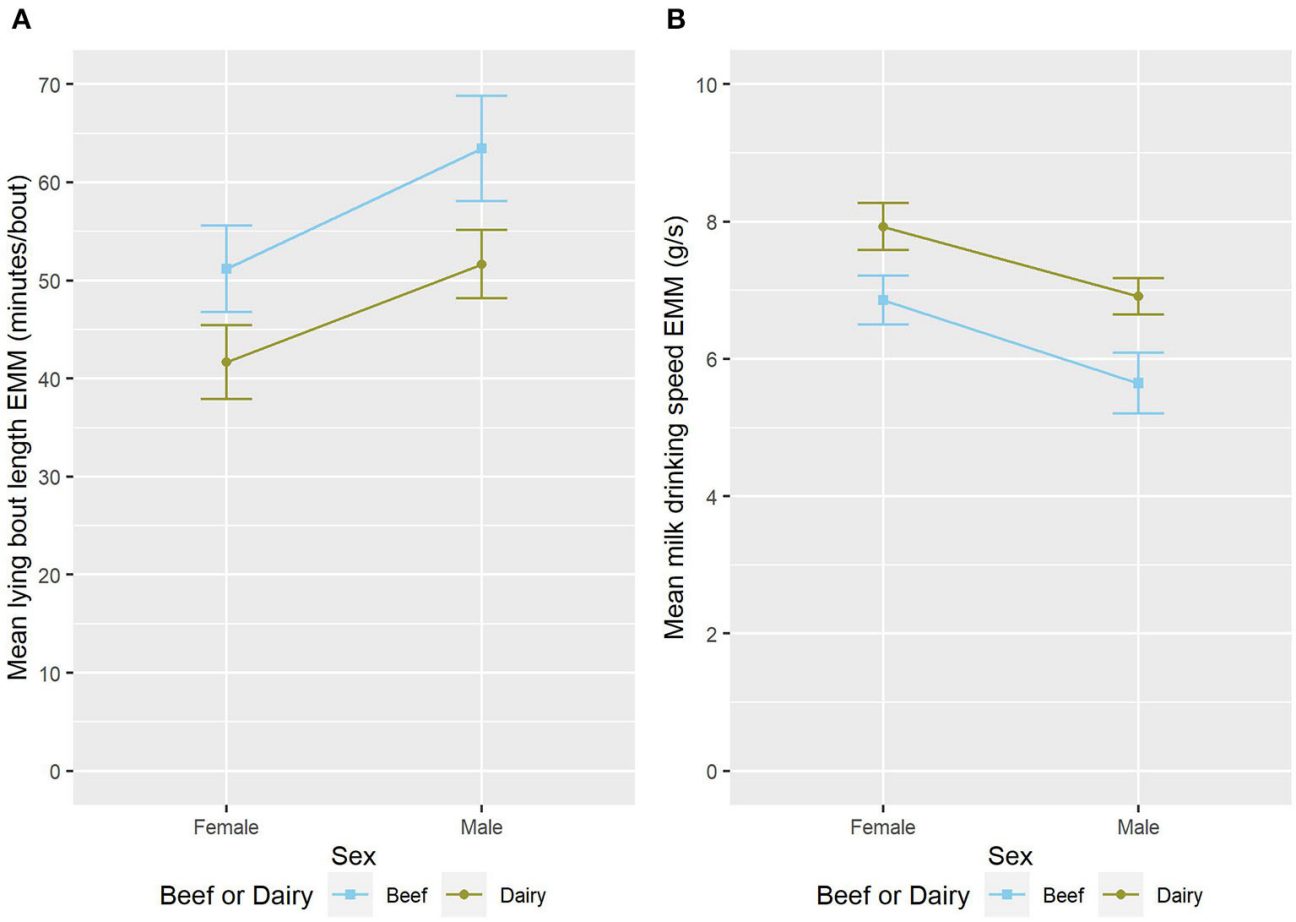
The estimated marginal means (EMM) of the **(A)** mean lying bout length and **(B)** mean milk drinking speed in relation to sex and sire breed type. Error bars denote the standard error.

Male calves had a longer mean lying bout length (ndf = 1, ddf = 22.5, *t* = 2.870, *p* = 0.009, Figure 4A) and tended to have fewer MI units/day than female calves (ndf = 1, ddf = 28.0, *t* = −1.919, *p* = 0.065, Figure 2B). Male calves tended to have longer daily lying time than female calves (ndf = 1, ddf = 26.3, Wald = 7.96, *p* = 0.071, **Table 4**, Figure 3A).

Female calves drink faster than their male counterparts (ndf = 1, ddf = 25.8, *t* = −3.033, *p* = 0.005, Figure 4B). Male calves were much less likely to consume their milk allowance (z = −2.530, *p* = 0.011, Table 3) than female calves. It is important to note that in this study live-weight was not significantly associated with sex when repeat measures and group were accounted for (results not shown).

**Table 3:** The effect of calf characteristics on the odds ratios of the total daily milk allowance having been drunk by healthy pre-weaned artificially reared calves.

**Fixed effect**	**Level**	**Odds ratio**	**95% Confidence interval**	***P*-value**
Sex	Female	Reference	Reference	Reference
	**Male**	**0.08**	**0.01 - 0.56**	**0.011**
**Live-weight**		**1.13**	**1.02 - 1.25**	**0.016**

Results shown are from the final binomial generalized linear mixed model output. Factors with *p* < 0.05 are shown in bold.

### 3.5. Factors with a significant but less profound association with calf behavior

**Figure 5:**
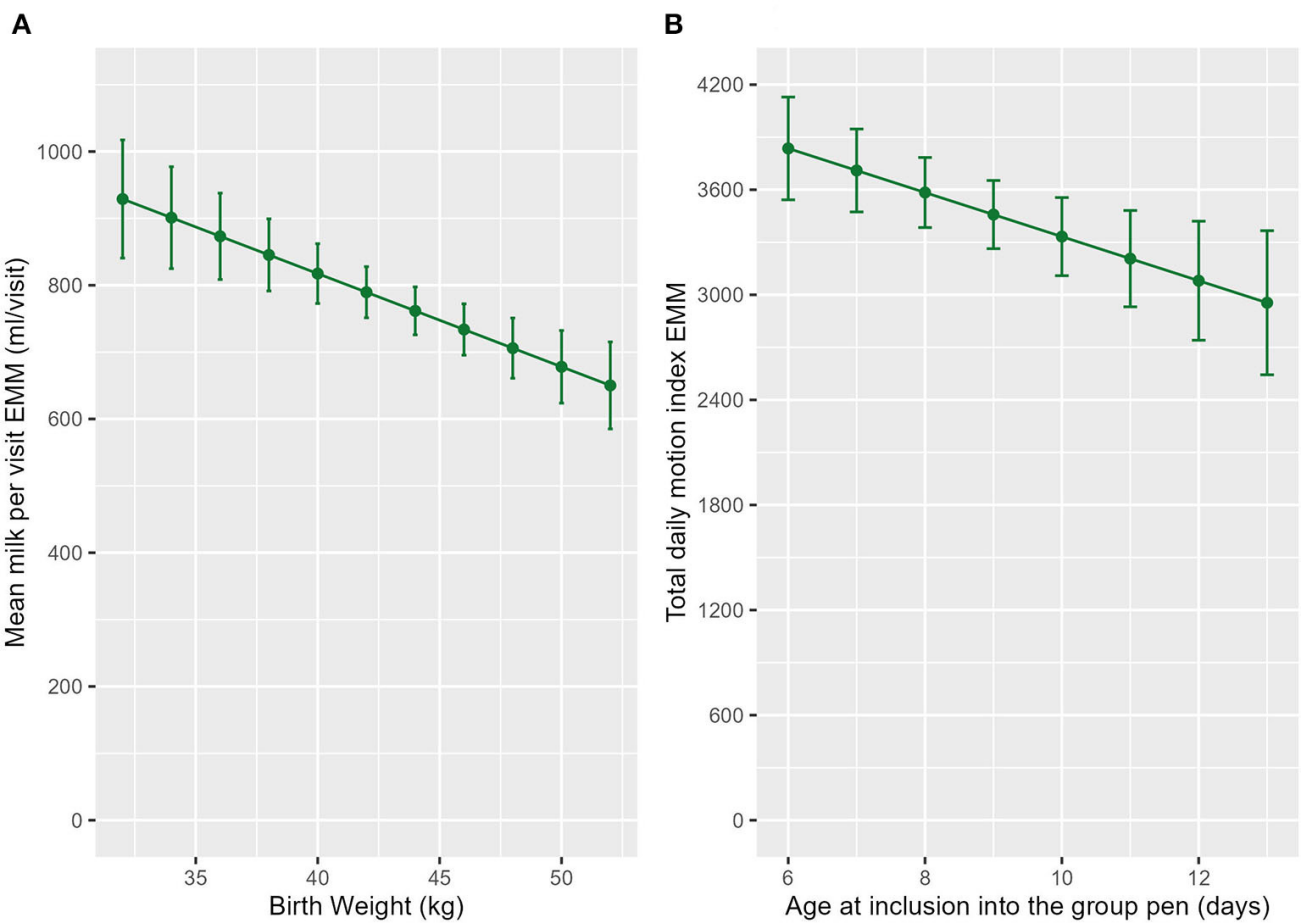
The estimated marginal means (EMM) of the **(A)** mean milk per visit in relation to birthweight. **(B)** EMM of the total daily motion index in relation to age at inclusion into the group pen. Error bars denote the standard error.

Calves that were born at a higher birthweight had fewer lying (ndf = 1, ddf = 25.2, *t* = −2.430, *p* = 0.023, Figure 3B) and standing (ndf = 1, ddf = 25.2, *t* = −2.462, *p* = 0.006, Supplementary Figure 1) bouts each day and a longer mean standing bout length (ndf = 1, ddf = 23.9, *t* = 2.217-, *p* = 0.002, Figure 3C). Calves that were born at a higher birthweight also drank less milk per feed (ndf = 1, ddf = 24.6, *t* = −2.154, *p* = 0.041, Figure 5A).

Compared to dairy-sired calves, calves with a beef sire had a longer mean lying bout length (ndf = 1, ddf = 21.9, *t* = −2.919, *p* = 0.008, Figure 4A). Beef-sired calves also drank more slowly (ndf = 1, ddf = 23.3, *t* = 3.359, *p* = 0.003, Figure 4B) than their dairy-sired counterparts.

Calves that were introduced into the group pen at an older age had a reduced MI per standing bout (ndf = 1, ddf = 28.0, *t* = −2.949, *p* = 0.007, Figure 5B). Age at inclusion into the group pen was also included in the final model for total MI but was not significant (ndf = 1, ddf = 26.9, *t* = −1.598, *p* = 0.121, Table 2).

Dam lactation was retained in the final model for mean milk per visit as it improved fit (ndf = 2, ddf = 24.2, F = 1.233, *p* = 0.309, Table 2) but was not significant in any other of the final models.

## 4. Discussion

This study found that live-weight, age, sex, and season of birth influenced activity and feeding behavior variables in pre-weaned artificially weaned calves. Birthweight, sire breed type and age at inclusion into the group pen did have significant associations but with fewer behaviors. Dam lactation had no association with behavior in pre-weaned calves.

The association of live-weight with healthy calf behavior has not previously been explored. However, some studies have used live-weight to pair diseased and healthy calves e.g., Duthie et al. (7) and included in disease prediction models e.g., Chisholm et al. (46). The current study found that heavier calves visited the milk machine more frequently (Figure 1A), for a shorter visit length (Figure 1B), drank faster (Figure 1C) and were slightly more likely to drink their milk allowance (Table 3) than their lighter counterparts. This concurs with a recent study that suggested a fast-drinking personality type is associated with increased live-weight gain (47). Another possible explanation is that due to the feed allowance being the same for all calves the larger calves were receiving a lower % of their bodyweight in milk and this was influencing their behavior. A previous study found time drinking was reduced in calves on a lower level of milk replacer (48). Calves fed less milk have been shown to visit the milk feeder more often (49).

Unlike in previous work, no increase in either the number of lying bouts (14) or daily standing time (15) with age was seen in this study. This study did, however, find that older calves had a lower total daily MI (Figure 2C), mean MI per standing bout (Figure 2B) and standing bout length (Figure 2A). This may reflect calves playing less as they get older. Calves have previously been found to have a reduction in play between 3 and 6 weeks of age (50). Another possible explanation is that stabilization of the group composition means that less movement is required. It may also reflect reduced exploration as calves become more familiar with their environment, for example, weaned calves visit different areas of the pasture more often when first introduced into a pasture but this behavior reduces over time (51).

Only one previous study has examined the association of birthweight with milk drinking behaviors and activity variables. It found that calves born at a normal (42.7 ± 2.6 kg) birthweight spent more time drinking milk and had a greater daily standing time than their lighter (34.9 ± 2.4 kg) counterparts (52). In contrast the current study found that calves that were born heavier had fewer lying (Figure 3B) and standing (Supplementary Figure 1) bouts each day, a longer mean standing bout length (Figure 3C) and drank less milk per feed (Figure 5B). However, in the current study the range of birthweights was greater (32–52 kg). The reduction in the number of lying and standing bouts was an interesting and unexpected finding, especially as birthweight is positively associated with play behavior in piglets (53). All the calves included in this analysis had an unassisted birth, so this association is unlikely to be due to dystocia. It is worth noting that birthweight in this study was influenced by both sex (*p* = 0.050, Supplementary Table 5) and dam lactation (first lactation-second lactation *p* = 0.013, first lactation- third lactation and over *p* = 0.002, Supplementary Table 5). In turn, birthweight had a significant effect on live-weight in a mixed model (*p* < 0.001, Supplementary Table 5). Interestingly a recent study found that as well as starting at a smaller size, calves with a smaller birthweight also grow more slowly (52). A larger study size may allow examination of interactions between the factors examined in this study, but this was not possible with only thirty-one calves.

Picking apart the associations between age, live-weight and birthweight and behavior is challenging. Live-weight is highly correlated with both age and birthweight (Supplementary Table 2). Interestingly, in Table 1 we can see that at the univariable level behaviors were only significantly (*p* < 0.05) associated with one or two of these three factors, this was unexpected due to the high level of correlation. The pattern seen in this study where when both age and weight had a *p* < 0.1 at the univariable stage, age produced a better fitting multivariable model for activity variables and weight produced a better fitting multivariable model for milk drinking behaviors was interesting. It is not however possible to pick out which of age and live-weight is more important.

Other studies have also recognized the importance of live-weight and age. For instance, both variables were previously included in random forest models for predicting bovine respiratory disease (54). It is worth noting that currently the inclusion of live-weight in predictive models for disease will require the automatic milk feeders to have a weighing platform, this is likely to require additional investment. Live-weight data also required significant data cleaning due to calves only partially standing on the scale or a second calf standing behind them. Thus, to use live-weight from a weighing platform in automatic disease detection tools automated cleaning of raw live-weight data prior to the disease detection model would be required or feeder design would need to be altered to ensure only a single whole calf could enter at any one time. Existing auto-weighers on the market already incorporate this data processing. An alternative would be to use a computer vision based system such as that used by Cominotte et al. (55) to estimate body weight. Using birthweight may be simpler and require less investment than live-weight. Thus, birthweight warrants further investigation as a possible alternative to live-weight for use in disease detection tools, possibly in conjunction with age.

In this study calves born in summer had a longer daily lying time (Figure 3A), more lying and standing bouts/day (Figure 3B and Supplementary Figure 1), and a shorter mean standing bout length (Figure 3C) than calves born in autumn or winter. It is highly likely that these associations are environmental in nature. The changes seen in the number of lying and standing bouts is consistent with the previous findings of differences in the number of posture changes in different levels of shade (22). The same study found no effect of shade on lying time however which does not concur with the finding that summer born calves had a longer daily lying time (Table 4) in the current study. The temperature and humidity index and the use of a ventilation system has been shown to influence the behaviors of 15 month old bulls (56), although the current study was conducted at far lower ambient temperatures (−5.5–28.2°C in current study compared to 20–30°C in bull study). Calves have been previously found to be aversive to wind (23) which may be reflected in the change in behavior in the autumn and winter. However, the association seen here is the opposite to what the authors would have expected. The associations with season in this study may not be completely due the conditions experienced by the calf. Calves born to cows that have been subject to heat stress in late lactation have been shown to have shorter standing bouts in the first week of life and shorter more frequent standing bouts in weeks seven and eight of life when compared to calves born to cows that had been cooled in late lactation (29). This is consistent with the findings of this experiment. This study has identified that season/environmental factors should be considered as it may alter behaviors. However, this analysis is limited by the fact that no calves included in this study were born between February and July and this should be considered for further studies. It may also be that future disease detection tools will also need to take into account factors such as substrate, which has been previously shown to affect locomotor play in an arena test (57).

**Table 4:** The effect of calf characteristics on the daily lying time in healthy pre-weaned artificially reared calves.

**Fixed effect**	**Wald statistic**	**Numerator degrees of freedom**	**F statistic**	**Denominator degrees of freedom**	***P* value**
**Season of birth**	**7.96**	**2**	**3.98**	**27.1**	**0.031**
Sex	3.54	1	3.54	26.3	0.071

Result shown are from the final multinomial generalized linear mixed model. Factors with *p* < 0.05 are shown in bold.

The finding of a tendency for increased activity (Total Daily MI) in female calves in this study concurs with a previous finding that female 6-week-old calves spend more time walking than their male counterparts (20) and may reflect the increased reaction to human approach previously seen in female calves (18). Interestingly, previous work has shown that male calves required fewer training sessions to use an automatic milk feeder than female calves (58). This contrasts with the finding in this study that male calves tended to drink slower (Figure 4B) and were much less likely to consume their daily milk allowance (Table 3). However due to the small sample size the interaction between sex and age was not tested so while this may be an effect of learning to use the feeder this cannot be confirmed.

Previous studies investigating effects of calf breed have not used either the same breeds or the same behaviors as the current study. The association of sire breed type with mean lying bout length (Figure 4A) and drinking speed (Figure 4B) seen in this study may reflect an effect of the calf's personality on their behavior. Previous authors have studied breed differences in some personality traits. For example, a study has previously shown that Holstein calves had a more fearful response in the escape test when compared to Holstein beef cross calves (17). Previous work using Holstein calves looked at three personality types, interactive, exploratory-active, and vocal inactive and found that more vocal calves have a greater number of rewarded visits to the feeder pre-weaning (59). It was not possible in this study to explore the interaction between breed and sex. This is due both to the small sample size and the skewed distribution between sex and breed. This occurred in part as an artifact of the data selection process and in part due to the breeding policies of the two sub-herds.

The association of age at inclusion into the group pen with mean MI per standing bout (Figure 5C) may reflect the reduction in social interaction seen in calves grouped at an older age (21). It is unlikely however that the change in behavior purely reflected the initial post grouping period however as calves were grouped at a maximum of 13 days of age (Mean 8.7 days, Supplementary Table 3) while the mean age of the data included in the final data set was 27.7 days. It was an interesting finding that age at inclusion into the group pen was not significant for any milk feeding behaviors in this study. Calves introduced to the automatic milk feeder at less than a day of age have previously been found to drink less milk in the first week of life than those fed three times daily by bottle until 5 days of age, however this effect was not seen after 8 days of age (58). This may explain why no association with milk feeding behaviors were seen in this study, as the days immediately after introduction to the group pen were not included. However, another study found that the frequency of rewarded visits was significantly lower in the first 12 days in the group pen in calves that were introduced at 6 days of age compared to those introduced at fourteen days of age, thus there may be an effect of age at introduction for a longer time period after introduction (60). Interestingly, in a study with a slightly lower age at inclusion into the group pen (mean 7.8 days, SD 1.9 days compared to mean 8.7 days, SD 2 days in this study) a higher age at inclusion was associated with an increase in the age at first calving (61).

Dam lactation was tested in this study as a proxy for maternal management as heifers will be managed differently to other cows. As the calves are removed as soon as possible after birth and are not fed colostrum from their own mother, differences in maternal behavior between lactations are unlikely to influence the calf. Lactation number was not associated with any of the behaviors in this study (Tables 2–4). This may be due to an insufficient difference in management between the lactation groups, especially in late pregnancy.

Several studies have examined changes in the number of unrewarded visits to the automatic milk feeder with disease. The number of unrewarded visits has been shown to decline in the 2 days prior to neonatal calf diarrhea diagnosis (62) and the 3 days prior to bovine respiratory disease diagnosis (63). Unfortunately, the automatic milk feeder used in this study did not record unrewarded visits to the automatic feeder. A potential area of further research is to repeat this experiment with an automatic milk feeder that records both rewarded and unrewarded visits to the feeder.

A further limiting factor of this study is the size of dataset. Thirty-one calves out of a possible 114 calves were included with ten calf days each. Gathering a sufficient data set of healthy calves is challenging due to the high incidence of disease in young calves. Across eleven UK dairy farms, on farm incidence of bovine respiratory disease and neonatal calf diarrhea in the first 9 weeks of life were 20.4–77.8% and 24.1–74.4%, respectively (2). While more relaxed inclusion criteria could have been used, the authors deemed that it was vital to remove any confounding caused by disease due to its strong effect on behavior. The small dataset is likely to particularly impact the investigation of the effect of lactation as studies into maternal factors would frequently analyze far larger datasets, e.g., 60 calves (29), 551 calves (64) or 15,992 calves (31). However, some studies have used a smaller number of calves, Ling et al. (25) used twelve calves in their study. Larger datasets are likely to be beneficial especially when attempting to identify subtle changes in behavior.

Interestingly, a recent study has shown that individual repeatability differs between milk feeding behaviors even after adjustment for age, cohort and day number (65). This could mean that further studies should focus on those behaviors with a high level of intra-individual repeatability. Intriguingly, feeding rate and the number of meals were highly correlated in a previous study (47) but were not significantly correlated in this study when correlations of the entire dataset were calculated (Supplementary Table 6), yet the approach of calculating correlations for individual calves and then an overall correlation may warrant further investigation.

In recent years the use of sensors and automated systems to detect disease has been increasingly researched (5). However, the performance of the reported models for bovine respiratory disease in pre-weaned calves is frequently poor, accuracies reported include 59.1% (66), 75% (54) and 80% (11). While no data is currently available on calf diseases, we know from previous work done on automatic tools for lameness detection in Canadian dairy herds that accuracy is a key concern for farmers (67). Farmers required a system that both detected a high proportion of lame cows and did not give many false alerts. Thus, it is vital that we have a thorough understanding of factors other than disease that are associated with changes in sensor measured behaviors in pre-weaned calves. This study aimed to address this gap in the literature and identified several factors that are associated with sensor measured behavioral variables. However, this study was conducted in a single shed; thus, it has its own environmental and management variables. To verify the results of this study the work needs to be repeated on several units.

## 5. Conclusion

This study found several intrinsic calf factors that are associated with healthy calf behavior. Most notable of these were live-weight, season, sex, and age. These are important considerations for comparing behavior between sick and healthy calves and predicting disease. However, there are likely to be farm level factors that also apply, and this needs further research as all calves in this study were reared in the same shed.

## Data availability statement

The raw data supporting the conclusions of this article will be made available by the authors, without undue reservation.

## Ethics statement

The animal study was reviewed and approved by Animal Experiment Committee of the Scottish Rural College.

## Author contributions

MH, C-AD, CM, JB, and DB designed the data collection of this work. BR developed the idea for the analysis, cleaned the data, performed the analysis, and wrote the first draft of the paper. MH, C-AD, AC, and CM provided support with the data cleaning and analysis. All authors assisted with drafting of the manuscript and approved the final manuscript.
