# Supplementary material for: Intrinsic calf factors associated with the behavior of healthy pre-weaned group-housed dairy-bred calves
Source: Front Vet Sci. 2023 Aug 3;10:1204580. doi: 10.3389/fvets.2023.1204580 (PMC10435862; doi:10.3389/fvets.2023.1204580)

Supplementary Material

1 Supplementary Data

Table 1 Sire breed type, sub-herd, and sex of the thirty-one calves included in the analysis.

**Dairy or beef**	**Sub-herd**	**Sex**	
		Male	Female
**Beef**	A	0	1
	B	5	5
**Dairy**	A	17	0
	B	0	3

Table 2 Correlation coefficients of three explanatory variables in thirty-one healthy pre-weaned calves

	Age	Live weight	Birth weight
Age	-	0.56	-0.04
Live weight	0.56	-	0.54
Birth weight	-0.04	0.54	-

Table 3 Descriptive statistics of possible explanatory variables in thirty-one healthy pre-weaned calves

	Mean	Standard Deviation	Minimum	Maximum	Standard Error of the Mean
Age (days)	27.7	6.0	11	39	0.3
Live Weight (Kg)	56.5	7.2	41.9	79.9	0.4
Birth weight (Kg)	43.6	5.2	32.0	52.0	1.0
Age at inclusion into the group pen (days)	8.7	2.0	6	13	0.4

Table 1 Descriptive statistics of feeding and activity behaviors in thirty-one healthy pre-weaned calves

	Mean	Standard Deviation	Minimum	Maximum	Standard Error of the Mean
Daily lying time (minutes)	973.7	63.7	679.2	1161.6	3.7
Daily standing time (minutes)	346.2	63.8	158.4	640.8	3.7
Daily lying bouts (n)	19.3	5.7	8.0	35.0	0.3
Daily standing bouts (n)	19.3	5.8	7.0	35.0	0.3
Total daily motion index	3328.5	1459.4	454.0	7445.0	83.8
Mean lying bout length (minutes)	55.3	18.2	22.4	139.3	1.0
Mean standing bout length (minutes)	19.6	7.3	5.6	54.4	0.4
Mean motion index per standing bout	177.1	75.6	32.4	470.1	4.4
Total time at milk (minutes)	15.8	3.5	4.5	35.6	0.2
Total milk visits (n)	8.5	2.2	2.0	16.0	0.1
Mean milk visit length (minutes)	1.8	0.6	1.1	4.3	0.0
Mean milk drinking speed (g/s)	6.8	1.4	1.0	10.4	0.1
Daily milk volume (ml)	6639.9	1012.9	1746.00	8416.0	57.5
Mean milk per feed (ml)	770.7	216.2	229.5	1380.8	12.3

Table 2 Final linear mixed effect model outputs showing factors affecting live weight and birthweight in thirty healthy pre-weaned calves. Factors with p<0.05 are shown in bold.

	Fixed effect	Level	Effect size	Confidence interval	P value
Live weight	Dairy or beef	Beef	Reference	Reference	Reference
		**Dairy**	**-3.5**	**-6.5- -0.5**	**0.032**
	**Age**		**0.8**	**0.8-0.8**	**<0.001**
	**Birth weight**		**0.8**	**0.5- 1.1**	**<0.001**
Birthweight	Sex	Female	Reference	Reference	Reference
		**Male**	**3.6**	**0.3- 6.9**	**0.0495**
	Dam Lactation	1	Reference	Reference	Reference
		**2**	**-7.2**	**1.5- 8.6**	**0.013**
		**3+**	**-6.7**	**2.9- 9.7**	**0.002**

Table 3 Correlation coefficients of milk drinking behaviors in thirty-one healthy pre-weaned calves

	Total time at milk	Total milk visits	Mean milk visit length	Mean milk drinking speed	Daily milk volume	Mean milk per feed
Total time at milk	-	0.52	0.22	-0.64	0.11	-0.38
Total milk visits	0.52	-	-0.54	-0.09	0.39	-0.70
Mean milk visit length	0.22	-0.54	-	-0.51	-0.50	0.57
Mean milk drinking speed	-0.64	-0.09	-0.51	-	0.54	0.32
Daily milk volume	0.11	0.39	-0.50	0.54	-	0.06
Mean milk per feed	-0.38	-0.70	0.57	0.32	0.06	-

Figure 1 The estimated means of daily standing bouts in relation to season and birthweight. Error bars denote the standard error.

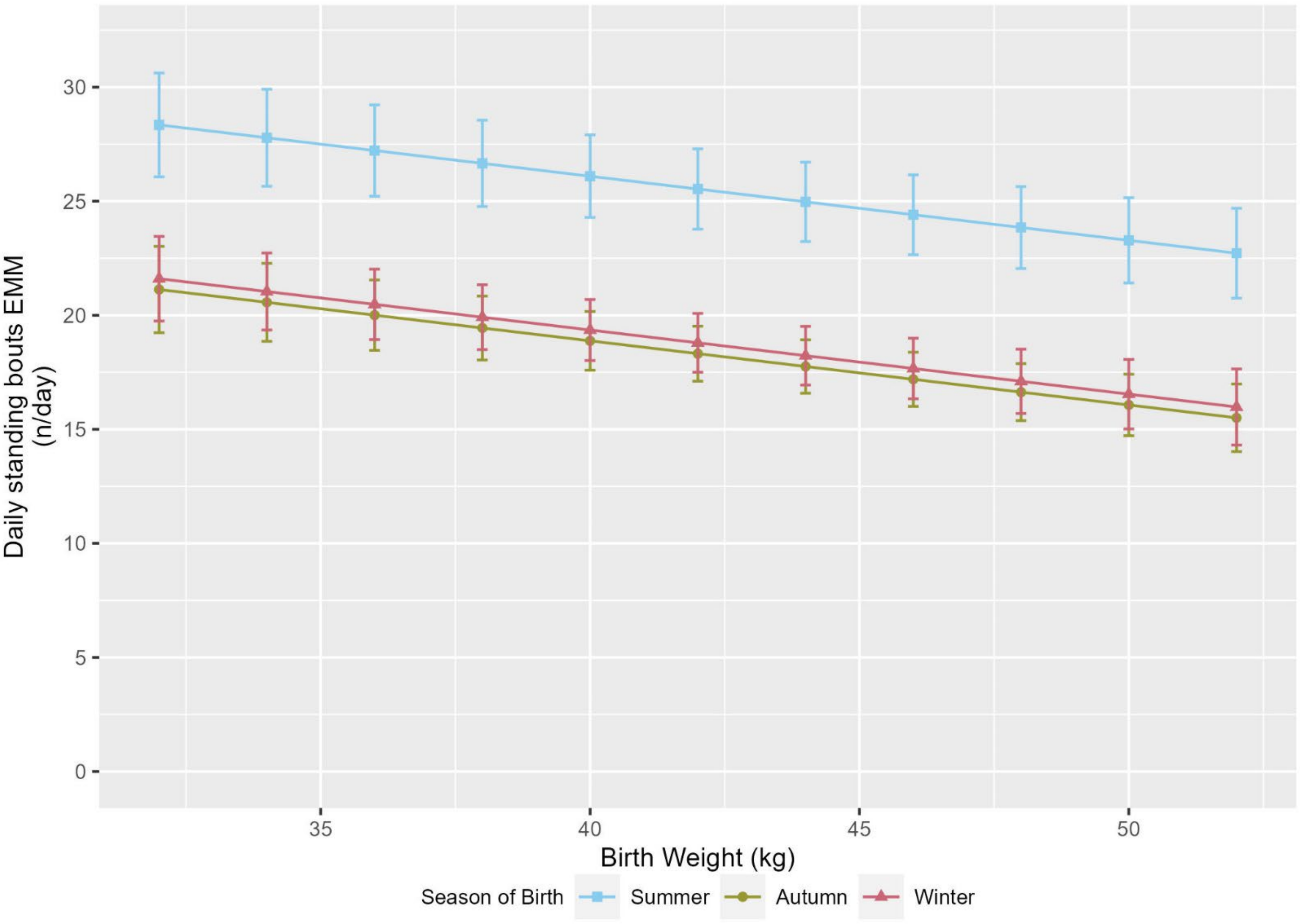

Supplement: Supplementary file 1 [file Data_Sheet_1.pdf]
